# Spectroscopic Detection of Glyphosate in Water Assisted by Laser-Ablated Silver Nanoparticles

**DOI:** 10.3390/s17050954

**Published:** 2017-04-26

**Authors:** Rafael Eleodoro De Góes, Marcia Muller, José Luís Fabris

**Affiliations:** Graduate Program in Electrical and Computer Engineering (CPGEI), Federal University of Technology-Parana, Curitiba 80230-901, Brazil; mmuller@utfpr.edu.br (M.M.); fabris@utfpr.edu.br (J.L.F.)

**Keywords:** glyphosate, silver nanoparticle, colorimetry, SERS

## Abstract

Glyphosate is one of the most widely used herbicides in the world. Its safety for both human health and aquatic biomes is a subject of wide debate. There are limits to glyphosate’s presence in bodies of water, and it is usually detected through complex analytical procedures. In this work, the presence of glyphosate is detected directly through optical interrogation of aqueous solution. For this purpose, silver nanoparticles were produced by pulsed laser ablation in liquids. Limits of detection of 0.9 mg/L and 3.2 mg/L were obtained with UV-Vis extinction and Surface Enhanced Raman spectroscopies, respectively. The sensing mechanism was evaluated in the presence of potential interferents as well as with commercial glyphosate-based herbicides.

## 1. Introduction

Glyphosate (*N*-(phosphonomethyl)glycine) is an effective broad-spectrum systemic herbicide successfully used worldwide for the control of undesirable weeds in cultures and gardens. The development of genetically modified crops resistant to glyphosate’s effects as well as its patent expiration have contributed to its wide use in different formulations [1]. Glyphosate can be found in a ready-to-use diluted formulation containing adjuvants and surfactants, as well as in concentrations as high as 48% v/v requiring correct dilution by the end user prior to the application.

Glyphosate is mainly absorbed through the leaves of the plants, and then it is transported throughout the plant inhibiting the action of enzymes responsible by the amino acid synthesis. This metabolic pathway in plants is absent in animals, justifying the low toxicity attributed for the glyphosate [1]. However, some studies have shown that glyphosate can also affect enzymes present in animals [2,3]. In spite of its low mobility in soil, whereas it is adsorbed onto soil particles, glyphosate can be taken by rain into water bodies, compromising the ecosystem, mainly aquatic plants and its food chain dependents. Some techniques have been proposed to minimize environmental impacts, as fish and aquatic invertebrates are more sensitive than mammals to glyphosate. In this sense, bacteria and metals have been suggested as mediators to promote glyphosate degradation [4,5].

The use of glyphosate represents a risk to human health that has been previously discussed, considering residuals of the herbicide found in food [6] and also the consequences of water contamination. Some works have focused on the carcinogenicity of glyphosate [7] whereas others have indicated that the increase in the hardness of ground water by the accumulation of metal-glyphosate compounds may be the cause of renal disease [8]. Controversies about its toxicity are a setback in establishing a safety limit for glyphosate by international agencies. In Brazil, for example, the presence of glyphosate in drinking water is limited to 500 µg/L (3 µM) whereas the limit set for the environment is 65 µg/L (0.4 µM) [9,10]. The Brazilian regulatory agencies carry out periodic analyses following norms such as method 547 established by the United States Environmental Protection Agency (US EPA draft 547) to ensure water conformity [11]. This water analysis demands expensive equipment and labored processes, usually column chromatography [12,13], as well as colorimetric methods associated with chemical reactions [14,15].

This bottleneck limits the geographic coverage and periodicity of the conformity tests, and therefore there is a demand for the development of highly sensitive, fast and cost-effective methods for the detection of glyphosate in water samples. In the last few years, nanotechnology has emerged as an alternative means for the detection of environmental pollutants due to its huge potential for overall cost reduction, reduced sample processing, as well as increased sensitivity [16].

Colorimetry has already been used for the detection of different substances in colloidal solutions of metal nanoparticles. In this technique, the controlled aggregation of the nanoparticles mediated by a specific analyte changes the characteristic extinction spectrum of the solution, allowing analyte detection. The position and intensity of the new Localized Surface Plasmon Resonance (LSPR) band are dependent on the chemical characteristics of the aggregation process. Such feature allowed the simultaneous detection of multiple analytes [17]. In some applications, the functionalization of the nanoparticle surface is used to add selectivity to the sensing process [18,19]. On the other hand, the Raman signature of the analyte can be identified by Surface Enhanced Raman Spectroscopy (SERS) of a colloidal solution of metallic nanoparticles [16]. SERS has the potential to overcome one of the most important limitations of the interrogation techniques based on Raman scattering: the low intensity of the signal resulting from the small scattering cross section, as well as the low concentration of the molecules under analysis [20].

The majority of the methods proposed in the literature depend on the fabrication of solid SERS substrates [21,22,23] although SERS is also achieved in colloidal solutions [24,25] of nanoparticles prepared by chemical (bottom-up) or physical (top-down) routes. Particularly for glyphosate detection, some authors have proposed SERS methods with solid substrates [21,26]. Colloidal functionalized gold nanoparticles solutions were also reported for glyphosate and other substances using only LSPR interrogation [15,24].

Metal nanoparticles from chemical route are produced basically by the reduction of metal salts in the presence of stabilizing agents. The attractive properties of this nanomaterials resulted in the establishment of many protocols for the formation of monodisperse solutions of nanoparticles with controlled shape and size that can be tuned for each application [27]. Nevertheless, despite the efforts, the experimental process still suffers from the absence of control. The morphology of the nanoparticles critically depends on the additives and stabilizing agents, concentration of metal salts and mainly on the crystallinity of the seeds [27,28].

On the other hand, Pulsed Laser Ablation in Liquids (PLAL) produces colloids of nanoparticles with heterogeneous distributions of size and shape. This feature can be used in the colloid preparation or in the interaction phase, for example by means of dimers formation [29], to promote the spectral tuning of the LSPR to a spectral region more suitable for the intensification of the Raman scattering. Besides, the already high field amplitudes close to the nanoparticles may experience further intensification for nanostructures presenting hotspots formed in the gaps produced by the colloidal destabilization that causes the nanoparticles’ aggregation [30]. In such a scheme, a Raman spectrum of glyphosate would result not only from the interaction with the substrate but also with the used solvent. Consequently, different spectra are expected with solid and liquid substrates. Using water as a solvent, broadened bands resulting from hydrogen bonds are observed, which are dependent on the pH of the solution [31].

Additionally, colloidal nanoparticles produced by PLAL can achieve a stable solution even with pure solvents and result in a less reactive surface than the chemically prepared ones [32,33]. Other advantages of the PLAL, if compared to the more common salt reduction methods, are its low cost and the absence of chemical waste. The desired colloidal solution destabilization can happen in the case the ζ potential, a measure of the surface charge of the particles in solution, falls below critical levels, leading to uncontrolled aggregation [19]. This can be achieved by changing the ionic strength with the addition of salts, or by modifying the pH by adding buffers to the solution. Another way to promote hotspot formation is a mediated aggregation where some ligand cross-link the nanoparticles, forming dimers, trimers or even higher order chains [34].

In this work, silver nanoparticles produced by PLAL in a citrate-water solution were used as substrate for the detection of glyphosate in water. The PLAL parameters and the proportion of citrate used as stabilizing agent were chosen to form a colloidal solution that remains stable during the measurement time span when glyphosate is added to the colloid. Two transduction principles were investigated: colorimetric, using the UV-Vis extinction spectrum of the LSPR that depends on the analyte concentration, and SERS that carries information not only about the analyte concentration but also about the interaction between the analyte and the colloidal silver substrate.

## 2. Materials and Methods

### 2.1. Silver Nanoparticle Production

**Figure 1:**
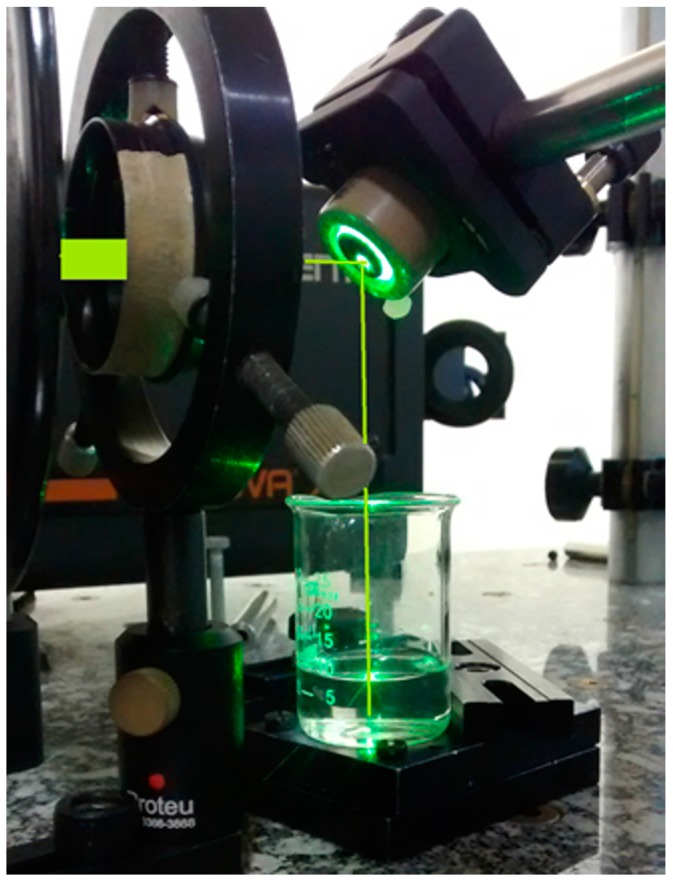
Picture of the experimental setup used for the production of the silver nanoparticles colloidal solution by Pulsed Laser Ablation in Liquid (the laser beam was highlighted).

Colloidal solutions of silver nanoparticles (AgNP) synthetized by PLAL were prepared from a silver foil (CAS number 7440-22-4, thickness of 1.0 mm, 99.9% trace metals basis) purchased from Sigma-Aldrich (product code 265543, St. Louis, MO, USA). A Nd:YAG laser (Tempest-20, New Wave Research, @532 nm, 5 ns pulse, 5 mm beam diameter, Fremont, CA, USA) was focalized by a 15 cm focal length lens on a Ag target at the bottom of a 25 mL-becker containing 10 mL of sodium citrate solution (Na_3_C_6_H_5_O_7_∙2H_2_O, analytical standard from Biotec) used as surfactant. This solution was prepared with deionized water (5 µS/cm conductivity) with a concentration of 0.1 mM of sodium citrate. The laser energy and pulse rate were adjusted to 17 mJ and 10 Hz, respectively, during an ablation time of 20 min. Figure 1 shows the experimental setup used for the nanoparticles synthesis.

In order to determine the morphology and atomic composition of the produced colloids, a drop of the colloidal solution was deposited on a glass-slide and left to dry during 24 h at room temperature. After that, the dried silver nanoparticles were covered with a 15 nm thick gold layer for scanning electron microscopy (SEM) imaging (Zeiss, EVO-MA15 equipped with Energy Dispersive Spectroscopy (EDS), Jena, Germany).

**Figure 2:**
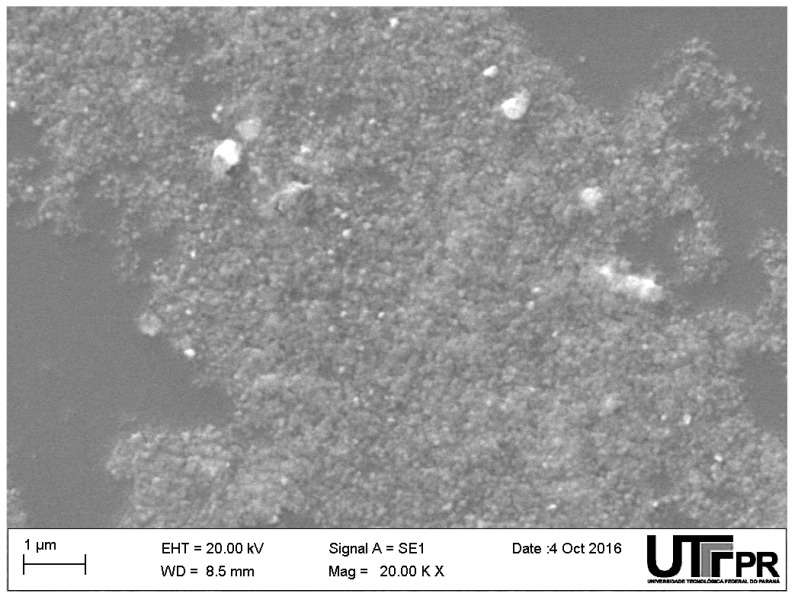
SEM micrograph of the dried colloidal silver nanoparticles from sample AgNP#0 (pure citrate capped silver nanoparticles).

The SEM image of the silver nanoparticles is shown in Figure 2. The image was taken in one of the borders of the so called “coffee-ring” effect [35]. The EDS analysis indicates the silver composition of the sample and the image reveals a broad size dispersion of spheroid particles with roughly estimated average diameter of 100 nm.

### 2.2. Analyte Samples

Samples of glyphosate and also samples containing some interferents were added to the colloidal solutions of silver nanoparticles submitted to the colorimetric and Raman techniques. Glyphosate (CAS number 10-71-83-6, linear formula (HO)_2_P(O)CH_2_NHCH_2_CO_2_H, analytical standard) was acquired from Sigma-Aldrich (product code 45521). To evaluate the performance of the detection scheme for interferents, glufosinate-ammonium salt (CAS number 77182-82-2), AMPA (aminomethil-phosponic acid, CAS number 1066-51-9), were also purchased from Sigma Aldrich (product codes 45520 and 05164, respectively) and sodium chloride (NaCl) was purchased from Biotec (Pinhais, Brazil). Aqueous solutions were prepared employing deionized water (5 µS/cm conductivity).

A primary solution of glyphosate at concentration of 6.5 g/L (40 mM) was diluted down to 4 mM by adding deionized water in the proportion 1:10. AMPA and glufosinate-ammonium were diluted to 4 mM, while sodium chloride was diluted to a concentration of 150 mM. Samples with different concentrations of analyte were prepared directly in disposable plastic cuvettes. First the cuvettes were filled with 0.9 mL of the AgNP colloid. Then, to each cuvette was added a chosen volume of one of the diluted solutions previously prepared. After that, the cuvettes were completed with deionized water to a final volume of 1.1 mL in order to maintain the same concentration of AgNP among the samples.

Samples containing two different commercial formulations of glyphosate, prepared according to the manufacturer instructions using tap water, were also analyzed. Each sample contains 200 µL of the commercial formulation added to 900 µL of the colloidal solution of AgNP. A sample containing AgNP and tap water spiked with analytical standard glyphosate was also prepared.

Table 1 lists the prepared samples and shows the analyte used in the formulation and their final concentration. The room temperature for the storage and measurements was maintained at 21.0 ± 0.5 °C. Standard Liquid Chromatography coupled to ion-trap Mass Spectroscopy (LC/MS) was employed for glyphosate content confirmation.

### 2.3. UV-Vis Measurements

**Figure 3:**
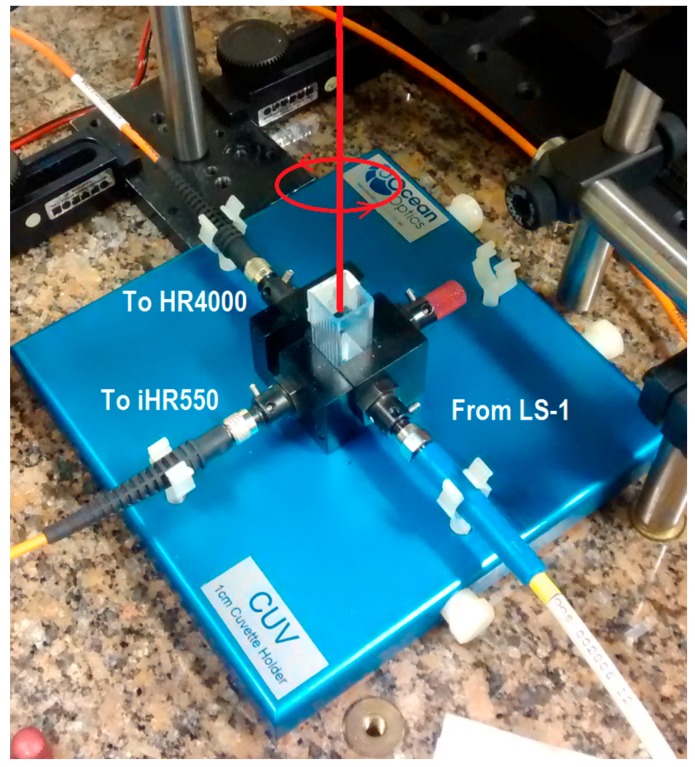
A picture of the sample holder of the experimental setup used for UV-Vis and Raman spectroscopic interrogation.

Figure 3 shows a detail of the experimental apparatus used to interrogate the liquid samples using UV-Vis as well as Raman spectroscopy. The optical source for UV-Vis extinction spectra (scattering plus absorption) of the colloidal samples is a tungsten halogen lamp (LS-1, Ocean Optics, Dunedin, FL, USA), coupled to the cuvette holder by a 500 µm core diameter optical fiber (Ocean Optics P200-2-UV-VIS). Light emerging from the cuvette is coupled to the spectrometer (Ocean Optics, HR4000, 200–1100 nm composite grating HC-1) by another 200 µm core diameter optical fiber (M25L02, Thorlabs, Newton, NJ, USA), resulting in a resolution of about 6.6 nm. The spectra were recorded using an integration time of 4 ms and 100 averages.

### 2.4. Raman Measurements

Light from a 632.8 nm He-Ne laser (Uniphase, 11335P, 9 mW power on the sample position, Milpitas, CA, USA) launched vertically on the sample (vertical red line in Figure 3) without any focalization was used as excitation source for the Raman scattering measurement. The laser beam is filtered by a 1 nm FWHM band pass filter (Thorlabs, FL05632.8-1) to remove the plasma lines and a λ/4 waveplate provides a circular polarization state (also depicted in Figure 3). Scattered radiation is collected perpendicularly to the incident beam and coupled by a 550 µm core diameter optical fiber (Thorlabs, M37L02) to the spectrometer (Horiba, Edison, NJ, USA, iHR550, 1200 g/mm grating blazed at 600 nm) equipped with a Charge Coupled Device detector (Synapse, 1024 × 256 elements) thermoelectrically cooled at −70 °C. The entrance slit is adjusted to 500 µm resulting in a 12 cm^−1^ resolution at 1600 cm^−1^. The spectra were measured with an integration time of 30 s and 4 averages.

## 3. Results and Discussion

### 3.1. Silver Nanoparticles with Glyphosate

The UV-Vis spectrum of the silver nanoparticles colloid (AgNP#0) shows a LSPR band centered close to 400 nm. The size of the capped silver nanoparticles is 16 nm as estimated by the peak position of the LSPR band. However, this approximation relies on a narrow distribution of sizes and considers the presence of a citrate layer but not the thickness of the silver oxide formed during the PLAL [32,36,37,38].

**Figure 4:**
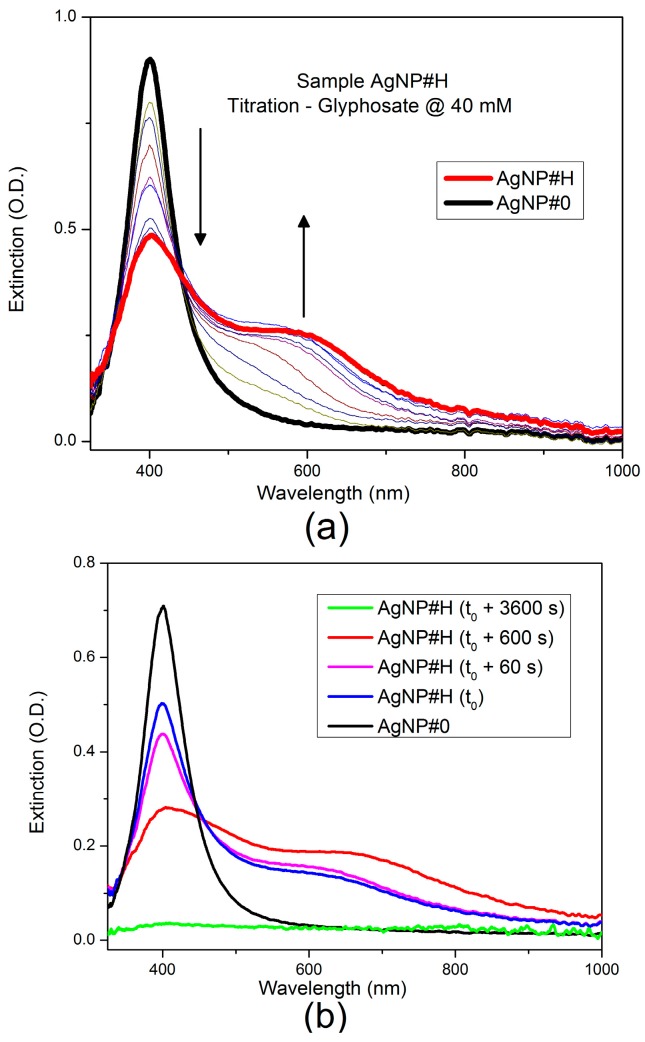

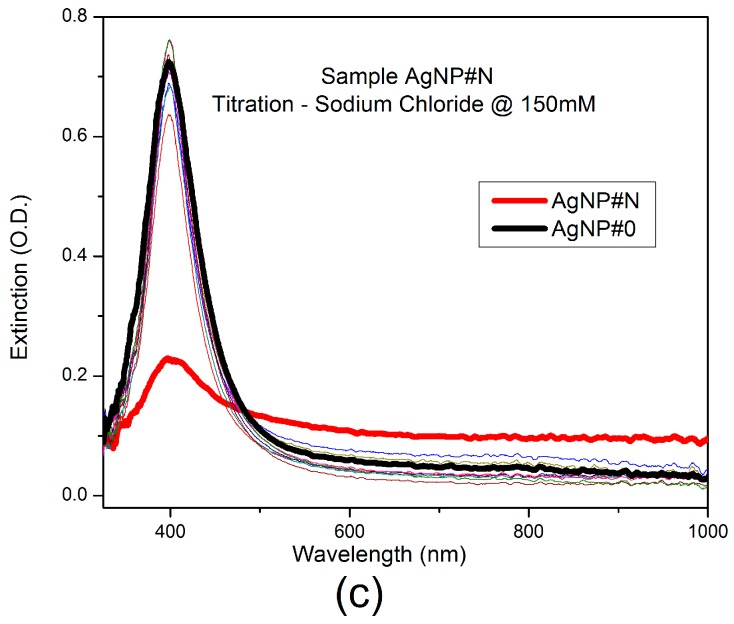
Extinction spectra of the AgNP colloidal solution (**a**) during titration with glyphosate at 40 mM (AgNP#H); (**b**) along the time for the sample with glyphosate concentration of 4 mM (AgNP#H); (**c**) during titration with sodium chloride at 150 mM (AgNP#N).

A preliminary analysis of the interaction between the AgNP colloid and glyphosate was carried out through a titration procedure in order to discriminate two concomitant effects, the glyphosate-mediated and the uncontrolled aggregation. Titration consisted in dripping 10 µL of the 40 mM primary solution of glyphosate into the cuvette filled with 0.9 mL of the AgNP colloid while recording the UV-Vis spectrum. The final glyphosate concentration achieved in the solution was 4 mM (AgNP#H). Figure 4a shows the spectra recorded with a time interval of 10 s.

The presence of glyphosate is associated with the appearance of a second LSPR band, at the expense of the original LSPR band at 400 nm. This second band is red-shifted relatively to the original one, indicating the aggregation and the formation of clusters of silver nanoparticles mediated by the glyphosate. A possible model assumes that the mediated agglomeration involves the hybridization of the plasmon modes of the interacting nanoparticles, resulting in higher energy (antibonding) or lower energy (bonding) plasmon modes [39].

Figure 4b shows the spectra obtained with the AgNP#H sample (4 mM of glyphosate) measured when it was produced, at the end of the titration process, and after 1, 10 and 60 min. This behavior shows that such a high concentration promotes the destabilization of the AgNP colloidal solution and the sedimentation of the metallic clusters at the bottom of the cuvette in a time span less than 1 h.

Figure 4c shows the effect of a titration with the primary solution of sodium chloride at 150 mM in the extinction spectrum of the AgNP colloid. In this case, the original AgNP LSPR band at 400 nm disappears after 10 min and new bands are not observed in the extinction spectrum at the final concentration of 4 mM. This behavior indicates the occurrence of a simple destabilization of the colloid due to the presence of sodium chloride.

**Figure 5:**
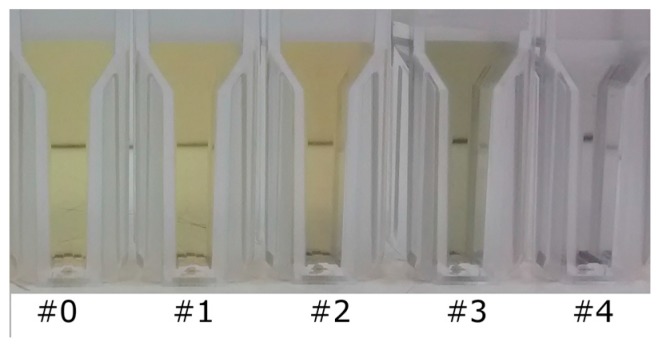
Picture of different colloidal solutions with increasing glyphosate content from left to right: AgNP#0 (pure citrate capped silver nanoparticles), AgNP#1 (120 µM of glyphosate), AgNP#2 (181 µM of glyphosate), AgNP#3 (243 µM of glyphosate) and AgNP#4 (545 µM of glyphosate).

Figure 5 shows the actual appearance of different colloidal solutions with increasing analyte concentrations 24 h after the production. As the concentration of glyphosate increases, the colloid color changes from yellow to dark green, as a result from the glyphosate-mediated nanoparticles aggregation. For glyphosate concentrations below 400 µM (samples AgNP#1, AgNP#2 and AgNP#3) the solutions presented pH values close to 5 and remained stable for more than a week.

**Figure 6:**
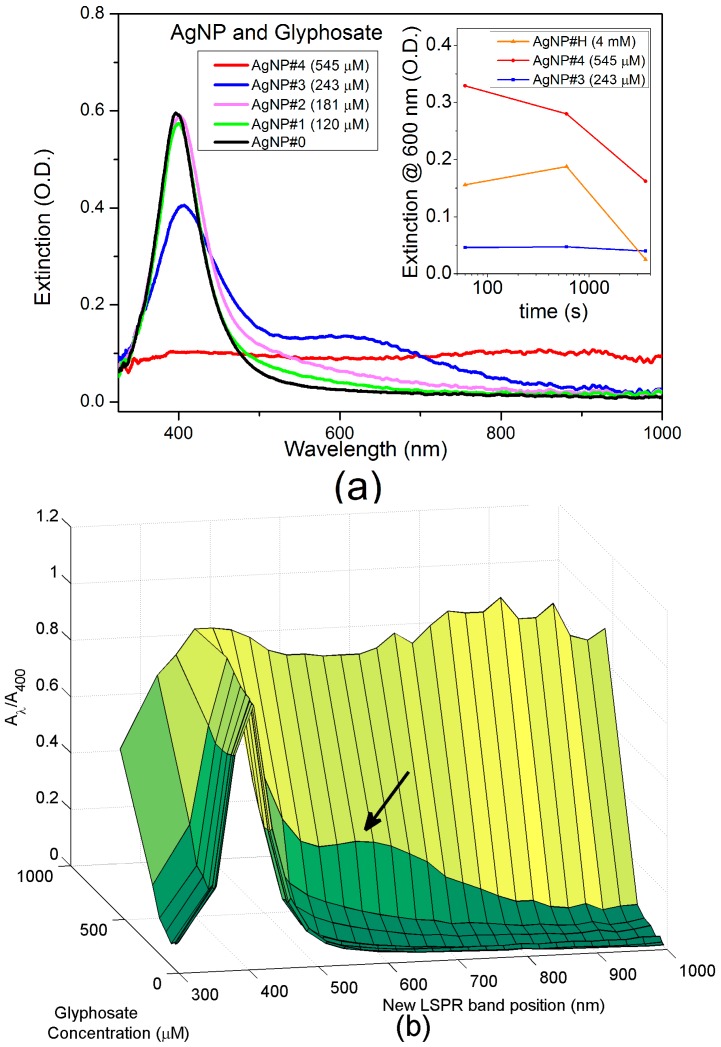
(**a**) UV-Vis extinction spectra of the samples with different concentrations of glyphosate. The inset shows the aggregation kinetics for three different concentrations; (**b**) LSPR extinction ratio as a function of the analyte concentration and the position of the new LSPR band. The arrow indicates the best response for the extinction ratio.

For concentration-sensitive experiments, UV-Vis extinction spectra of four samples (AgNP#1, AgNP#2, AgNP#3 and AgNP#4) were measured 3 h after the preparation. The obtained results are shown in Figure 6a. Colloid destabilization and uncontrolled aggregation occurs for glyphosate concentrations higher than 400 µM. The inset of Figure 6a shows the aggregation kinetics for three different concentrations. Among the three samples, only the AGNP#3 with 243 µM of glyphosate is stable. Such processes produce, instead of a new well defined LSPR band, the long tail characteristic of Rayleigh scattering from larger agglomerates as the surface plasmon is no longer localized. A deposit of silver aggregates is formed at the bottom of the cuvette for the sample with the highest concentration of glyphosate (AgNP#4, 543 µM) in less than 24 h. Given that, for the parameters used in the PLAL synthesis, the highest concentration of glyphosate for the production of a stabilized solution is around 400 µM. Figure 6b shows a surface chart that relates the extinction ratio (A_λ_/A_400_) as a function of the analyte concentration and the new LSPR band central wavelenght, A_λ_. It is noticed by the peak at the “new LSPR band position” axis and by the increased slope of the “glyphosate concentration” axis that the best response to the glyphosate presence occurs at 600 nm, as indicated by the arrow in Figure 6b.

**Figure 7:**
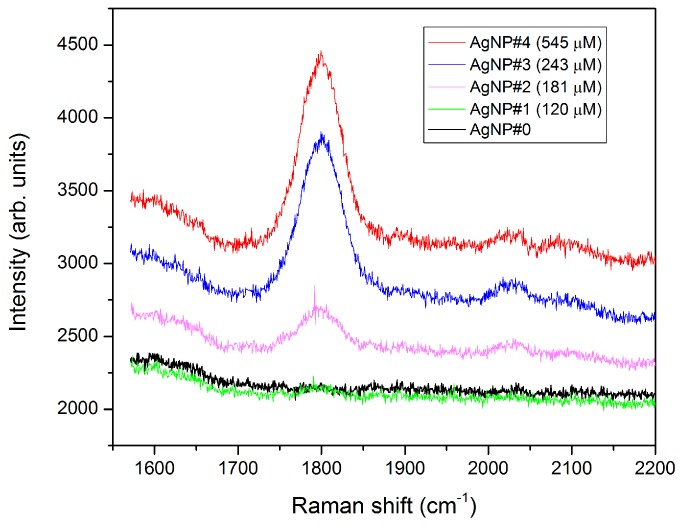
Raman spectra of AgNP colloidal solutions with different glyphosate concentrations.

Complementary information about the interaction between the analyte and the AgNP colloid was obtained by measuring the Raman scattering spectrum. Figure 7 shows the Raman spectra of the samples with glyphosate concentrations from 0 to 540 µM. The band close to 1800 cm^−1^ with a bandwidth of 50 cm^−1^ can be tentatively assigned to the asymmetric vibration of the OCCO group, more specifically to CO double bond [40]. The high intensity of the scattering may be associated with the conversion from the form I of glyphosate (zwitterion) to the monoanion, when the solution pH varies from 4 to less than 8, and the subsequent interaction with the negatively charged AgNPs capped with citrate anions. The intensity of the Raman scattering in this band depends on the glyphosate concentration. For concentrations higher than 400 µM a complete colloid destabilization occurs in less than 24 h, as shown in Figure 5. It can be asserted that in this case, for 3 h interaction time, the dimers are still in suspension, whereas the larger agglomerates have already deposited, as can be seen from the comparison between the Raman and UV-Vis spectrum obtained at the same time.

SERS interrogation of colloidal solutions has some advantages when compared with the interrogation of solid substrates. In spite of the fact that the SERS signature is blurred by non-radiative transitions and hydrogen bonding with the water matrix, the interrogation is taken in an ensemble volume. This aspect somewhat circumvents repeatability problems in the production of dried substrates for deposition and interrogation with Raman microscope. Interrogation of a solution, without changing the matrix to another solvent as in the case of liquid chromatography, is advantageous for the water quality monitoring. It opens up the possibility of using microfluidic interrogation techniques for on-line verification [41] and has the potential to be a complementary pre-compliance analysis technique that can be used for the quality assurance of water resources.

### 3.2. Silver Nanoparticles with Interferents and Comercial Formulations

**Figure 8:**
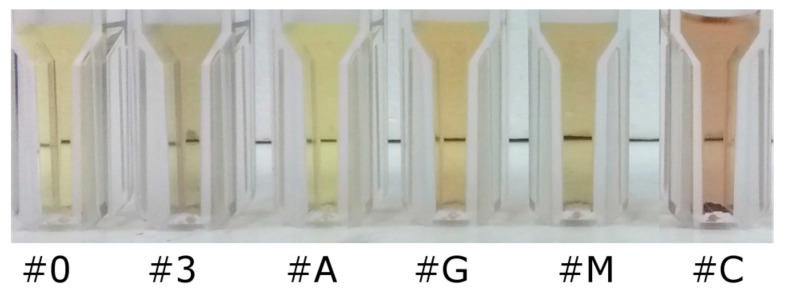
Picture of the samples: AgNP#0 (pure citrate capped silver nanoparticles); AgNP#3 (243 µM of glyphosate); AgNP#A (243 µM of AMPA); AgNP#G (243 µM of glufosinate-ammonium); AgNP#M (300 µM of glyphosate in commercial formulation 1) and AgNP#C (300 µM of glyphosate in commercial formulation 2).

Figure 8 shows a picture of the solutions containing the potential interferents AMPA and glufosinate-ammonium as well as two commercial glyphosate formulations.

**Figure 9:**
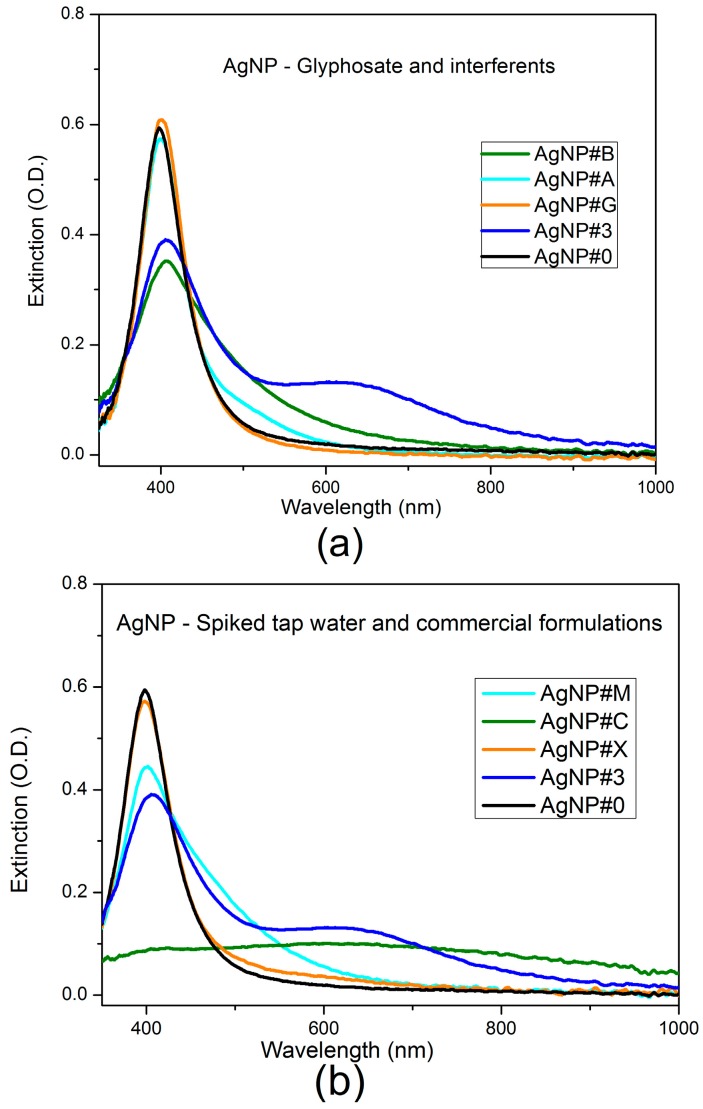
UV-Vis extinction spectra of: (**a**) interferents; (**b**) commercial formulations. For both cases, pure citrate-capped silver nanoparticles (AgNP#0) and citrate-capped silver nanoparticles with 243 µM of glyphosate (AgNP#3) are shown as reference. Samples: AgNP#B (tap water), AgNP#A (243 µM of AMPA), AgNP#G (243 µM of glufosinate-ammonium), AgNP#M (300 µM of glyphosate in commercial formulation 1), AgNP#C (300 µM of glyphosate in commercial formulation 2), AgNP#X (tap water with glyphosate at final concentration of 243 µM).

The UV-Vis extinction spectra of Figure 9 show that the simple colorimetric transduction scheme can be influenced by the concomitant effect of unmediated colloid destabilization triggered by the investigated interferents.

The LSPR band at 600 nm only appears with citrate capped AgNP produced by PLAL in the presence of glyphosate. The occurrence of the long tail effect without the generation of a new LSPR band, characteristic of scattering by larger nanoparticle clusters, can affect the measurement if a simple approach of extinction ratio at 600 nm by 400 nm is taken. Such an approach is commonly employed in colorimetric sensing with functionalized nanoparticles. This behavior is more pronounced for two commercial glyphosate samples (AgNP#C and AgNP#M) with formulations containing unspecified surfactants.

**Figure 10:**
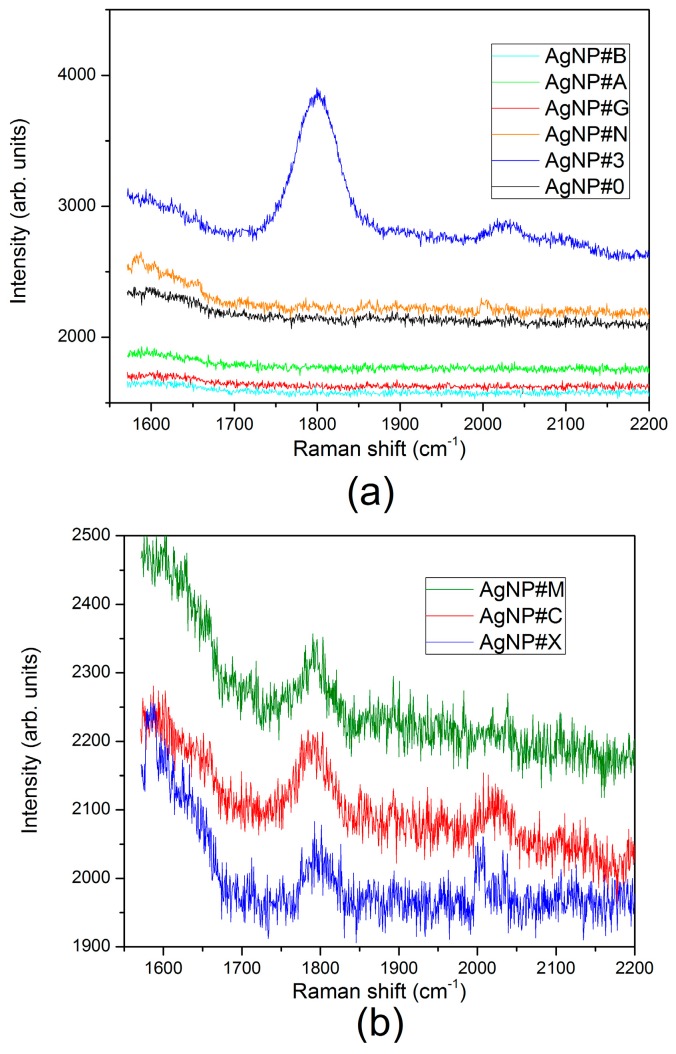
Raman scattering spectra of: (**a**) interferents; (**b**) commercial formulations (the spectra were shifted vertically for better visualization). For both cases, pure citrate-capped silver nanoparticles (AgNP#0) and citrate capped silver nanoparticles with 243 µM of glyphosate (AgNP#3) are shown as a reference. Samples: AgNP#B (tap water), AgNP#A (243 µM of AMPA), AgNP#G (243 µM of glufosinate-ammonium), AgNP#N (4 mM of sodium chloride), AgNP#M (300 µM of glyphosate in commercial formulation 1), AgNP#C (300 µM of glyphosate in commercial formulation 2), AgNP#X (tap water with glyphosate at final concentration of 243 µM).

Raman spectra of potential interferents and commercial formulations were also measured and the results are shown in Figure 10. It can be seen that the band at 1800 cm^−1^ is only present in the spectra of the samples that contains glyphosate. However, the amplitude of this band in the samples of commercial formulations AgNP#C, AgNP#M and spiked tap water AgNP#X is lower than that expected for the actual concentrations. This quantification misreporting could be attributed to the formulation that employs surfactants to ease the application in the field as well as to impurities in the tap water.

**Figure 11:**
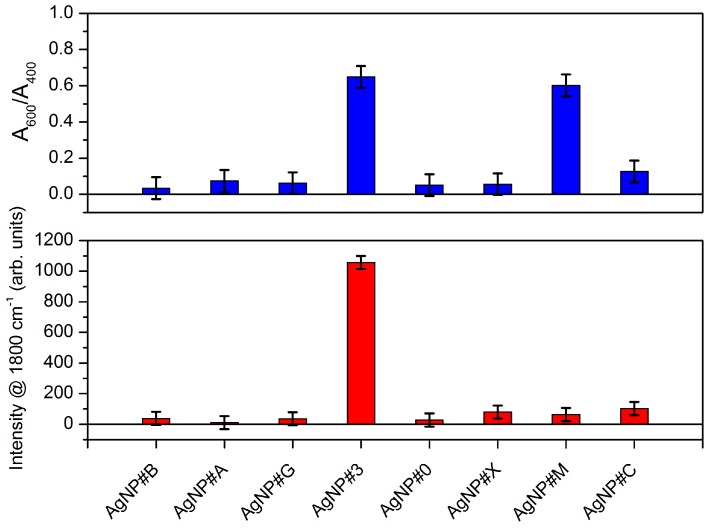
Glyphosate and interferent detection using the two transduction mechanisms. Samples: AgNP#B (tap water), AgNP#A (243 µM of AMPA), AgNP#G (243 µM of glufosinate-ammonium), AgNP#3 (243 µM of glyphosate), AgNP#0 (pure citrate-capped silver nanoparticles), AgNP#X (tap water with glyphosate at final concentration of 243 µM), AgNP#M (300 µM of glyphosate in commercial formulation 1), AgNP#C (300 µM of glyphosate in commercial formulation 2).

Figure 11 shows a summary of the results obtained with the two sensing methods based on the interaction of the analytes with the AgNP colloidal solution. For the colorimetric method, quantification can be done using the ratio between the extinction of different LSPR bands. For the SERS of the colloidal solution, the intensity of the band at 1800 cm^−1^ relates to the concentration. Based on the IUPAC (the International Union of Pure and Applied Chemistry) recommendations [42,43], the limit of detection (LOD) for β = 0.05 (the probability of false negative) and a constant standard deviation (σ_0_) of the estimate analyte amount or concentration when its true value is zero is LOD = 3.29 × σ_0_. From the UV-Vis extinction ratio, the resulting limit of detection is about 0.9 mg/L (5.4 µM), whereas, for the Raman spectra, this limit is 3.2 mg/L (19 µM).

With respect to the specificity, the detection based in the UV-Vis spectrum of the LSPR band seems to be more prone to interferents that could act in the aggregation process, as is the case for the AgNP#M sample. The combination of both interrogation mechanisms in the same sample may lead to gains in selectivity as well as in specificity.

**Figure 12:**
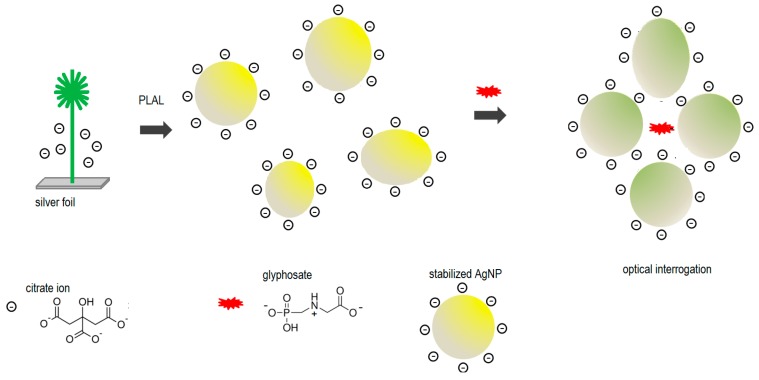
Schematic representation of the glyphosate sensing mechanism with citrate-capped colloidal AgNP.

Regarding the mediated aggregation, a possible interpretation is that glyphosate promotes the aggregation of the nanoparticles that are kept stable by the easily displaceable citrate anions that form the electric double layer on the surface of the AgNP. This behavior is proposed since the measurements taken, even in the case of destabilization of the colloidal solution, show the appearance of the enhanced Raman band at 1800 cm^−1^ and the second LSPR band at 600 nm only in the presence of glyphosate. This scheme is presented in Figure 12. The broad distribution of particle size resulting from the PLAL synthesis route can help in ensemble hot spot formation [29].

## 4. Conclusions

A sensing approach to detect glyphosate in water samples assisted by citrate-capped silver nanoparticles was presented. For this approach, a colorimetric transduction which uses the ratio between extinction bands and also a transduction with higher specificity using the SERS bands are shown. Tests carried out with interferents and commercial glyphosate formulations indicate that both UV-Vis and SERS spectra have features that depend on the glyphosate concentration. Results from this work creates the opportunity for the investigation of methods for the simultaneous chemometric evaluation of data obtained by the UV-Vis and SERS interrogation. Additionally, the determination of the ideal pH of the solution and PLAL parameters would be used in order to achieve a better sensitivity and dynamic range. Although the limits of detection achieved with both techniques are above the safety limit established by most international agencies, the proposed approach can be useful for a pre-qualification of samples regarding the presence of glyphosate.

## Figures and Tables

**Table 1:** Samples prepared in disposable plastic cuvettes with final volume 1.1 mL and optical path of 4.5 mm.

Sample	Concentration (µM)	Analyte
AgNP#0	0	-
AgNP#1	120	Glyphosate
AgNP#2	181	Glyphosate
AgNP#3	243	Glyphosate
AgNP#4	545	Glyphosate
AgNP#H	4000	Glyphosate
AgNP#N	4000	Sodium chloride
AgNP#A	243	AMPA
AgNP#G	243	Glufosinate-ammonium
AgNP#M	300 ^1^	Commercial formulation 1
AgNP#C	300 ^1^	Commercial formulation 2
AgNP#B	0	Tap water
AgNP#X	243	Tap water with glyphosate

^1^ As estimated considering the content indicated by the manufacturer and the dilution of the solution.
